# A Systematic Review of Efficacy, Safety, and Tolerability of Duloxetine

**DOI:** 10.3389/fpsyt.2020.554899

**Published:** 2020-10-23

**Authors:** Daniela Rodrigues-Amorim, José Manuel Olivares, Carlos Spuch, Tania Rivera-Baltanás

**Affiliations:** ^1^Translational Neuroscience Research Group, Galicia Sur Health Research Institute (IISGS), University of Vigo, Centro de Investigación Biomédica en Red de Salud Mental (CIBERSAM), Vigo, Spain; ^2^Head of Department of Psychiatry, Health Area of Vigo, Servizo Galego de Saúde (SERGAS), Vigo, Spain; ^3^Director Neuroscience Area, Galicia Sur Health Research Institute (IISGS), Centro de Investigación Biomédica en Red de Salud Mental (CIBERSAM), Vigo, Spain; ^4^Translational Neuroscience Research Group, Galicia Sur Health Research Institute (IISGS), Centro de Investigación Biomédica en Red de Salud Mental (CIBERSAM), Vigo, Spain

**Keywords:** duloxetine, clinical conditions, efficacy, safety, tolerability

## Abstract

Duloxetine is a serotonin-norepinephrine reuptake inhibitor approved for the treatment of patients affected by major depressive disorder (MDD), generalized anxiety disorder (GAD), neuropathic pain (NP), fibromyalgia (FMS), and stress incontinence urinary (SUI). These conditions share parallel pathophysiological pathways, and duloxetine treatment might be an effective and safe alternative. Thus, a systematic review was conducted following the 2009 Preferred Reporting Items (PRISMA) recommendations and Joanna Briggs Institute Critical (JBI) Appraisals guidelines. Eighty-five studies focused on efficacy, safety, and tolerability of duloxetine were included in our systematic review. Studies were subdivided by clinical condition and evaluated individually. Thus, 32 studies of MDD, 11 studies of GAD, 19 studies of NP, 9 studies of FMS, and 14 studies of SUI demonstrated that the measured outcomes indicate the suitability of duloxetine in the treatment of these clinical conditions. This systematic review confirms that the dual mechanism of duloxetine benefits the treatment of comorbid clinical conditions, and supports the efficacy, safety, and tolerability of duloxetine in short- and long-term treatments.

## Introduction

Depression and chronic pain are disabling and often concomitant pathologies; both are currently two of the main public health problems (1, 2). Major depressive disorder (MDD) is the most prevalent psychiatric disease and has been recognized as a critical target of intervention in the psychiatric field (3, 4). However, depression remains underdiagnosed and consequently, undertreated (4, 5). Furthermore, generalized anxiety disorder (GAD), obsessive-compulsive disorder (OCD), and post-traumatic stress disorder (PTSD) are common psychiatric comorbidities with MDD, usually lead to worse prognosis and compromise the remission of MDD symptoms (6).

GAD is one of the most prevalent psychiatric disorders, affecting 6% of the population during their lifetime (7, 8). GAD is a chronic condition that severely affects the quality of life, due to its repercussion on working and social functioning (8). Even though anxiety is a widespread symptom, the diagnosis of GAD requires a complex process of screening for a correct diagnosis (9). Moreover, GAD is usually associated with other clinical conditions such as MDD or pain syndromes, affecting drastically prognosis, and treatment efficacy (10).

Chronic pain is a persistent pain condition with a dual dimension, based on the signaling mechanism pathways: nociceptive and neuropathic pain (NP) (11). Specifically, NP has a strong emotional implication, and has been associated with worse quality of life, and clinically, it is related with affective disturbances such as depression, anhedonia, working memory dysfunction, sleep disturbances, anxiety, and impaired cognition (12–14). Moreover, chronic pain involves a stress component that might play a bidirectional predictive role. That is, chronic stressful events produce biochemical and pathophysiologic alterations that lead to stress-related mood disorders, that also may increase the risk of chronic pain or exacerbate it (14, 15).

On the other hand, fibromyalgia syndrome (FMS) is a chronic widespread pain condition with high heterogeneity clinically and etiologically (16, 17). It is estimated that 4–6% of adults worldwide suffer from FMS, whose incidence is increased in women (18). The most debilitating symptom of FMS is generalized pain. Other symptoms such as fatigue, sleep disturbances, cognitive impairment, or headache are also part of the core symptoms of FMS (19). Concomitantly, MDD symptoms also overlap with the FMS, as well as GAD that is significantly higher in patients with FMS (20, 21).

There is a possible connection between anxiety, depression, and stress urinary incontinence (SUI). Evidence suggests that both anxiety and depression are predictor of SUI onset (22). SUI is characterized by an unintentional urinary leakage due to coughing, exertion or sneezing, which increase the intra-abdominal and bladder pressure that overcome urethral resistance (23). Serotonin (5-HT) pathways are involved in this disorder. Thus, 5-HT induces the urethral sphincter closure by inhibition of the micturition reflex (22).

In this perspective, duloxetine is a potential treatment for these dissimilar clinical conditions, but with shared pathophysiological pathways. Duloxetine is a serotonin-norepinephrine reuptake inhibitor (SNRIs) approved as a first-line drug to treat MDD, GAD, diabetic peripheral neuropathy (DPN), FMS and SUI (24–28). As a SNRIs, duloxetine increases both levels of serotonin and norepinephrine which are directly correlated with adverse events, such as tachycardia, hypertension, among others (29). Pharmacokinetic and pharmacodynamic data of duloxetine have been reported for several studies, whose evidence suggests that duloxetine is generally well-tolerated (30–33). Thus, the main goal of this systematic review was to determine the efficacy, tolerability, and safety of duloxetine in the treatment of the clinical conditions for which it is approved.

## Methodology

### Study Design

A qualitative systematic review of literature was performed, following the 2009 Preferred Reporting Items for Systematic Reviews and Meta-Analysis (PRISMA) guidelines, and the Joanna Briggs Institute (JBI) critical appraisal checklist for the different types of studies reviewed (34). This systematic review aimed to describe and synthetize the evidence and potential benefits of duloxetine.

### Protocol Registration

The protocol was registered in the international database PROSPERO of the National Institute for Health and Research (NIHR) with the code CRD42020153634.

### Eligibility Criteria

To accomplish this comprehensive systematic review the following inclusion criteria were assumed: all studies written in English and focused on human adults (at least 18 years old) with MDD, GAD, NP, FMS, or SUI (clinical conditions for which duloxetine has approval), published until 01/09/2020. Those studies whose primary outcomes were efficacy, tolerability, and/or safety of duloxetine were included. Studies focused on other psychiatric or neurological condition such as Parkinson's disease, Alzheimer's disease, chronic non-neuropathic pain, bipolar, schizoaffective, and schizophrenia disorders were excluded. Moreover, qualitative research reports were also excluded, as well as reports whose analyses were based on pooled integrative data analysis of randomized control trials (RCTs). Eligible designs included RCTs, non-randomized experimental studies, case-control, and cohort studies, which outcomes were quantitatively measured by social, functional, cognitive, quality of life (QoL), or treatment emergent adverse events (TEAEs) instruments.

### Data Sources and Search Strategy

Studies were selected from PubMed, Medline, Web of Science, and PsycINFO electronic databases, introducing the search terms: “duloxetine” AND “major depressive disorder” OR “MDD”; “duloxetine” AND “generalized anxiety disorder” OR “GAD”; “duloxetine” AND “neuropathic pain”; “duloxetine” AND “fibromyalgia”; and “duloxetine” AND “stress urinary incontinence.” Two independent researchers (DRA and JMO) conducted the search strategy, applying the filters described in the inclusion criteria to refine the process and obtain concise results.

### Study Selection

Authors independently screened the reports. Firstly, titles and abstracts were reviewed to evaluate their concordance with our requirements. Secondly, the full-text of the potential studies were screened and appreciated and those that met our inclusion criteria were selected. Finally, 85 studies were included in this systematic review. Discrepancies were resolved through discussion among the authors until consensus was reached.

### Data Extraction and Synthesis

To summarize the relevant information of the selected studies, the authors extracted and performed a Table with the following data: first author and year of publication, number of participants, gender, mean age (years), duloxetine dose per day (mg), duration of the treatment (weeks), diagnosis scales or other clinical measuring instruments, relevant statistical results, type of study, and the principal outcomes. The process of synthesis allowed a critical appraisal of the studies and the effect size calculation based on the statistical data reported by studies.

## Results

### Search Results

**Figure 1:**
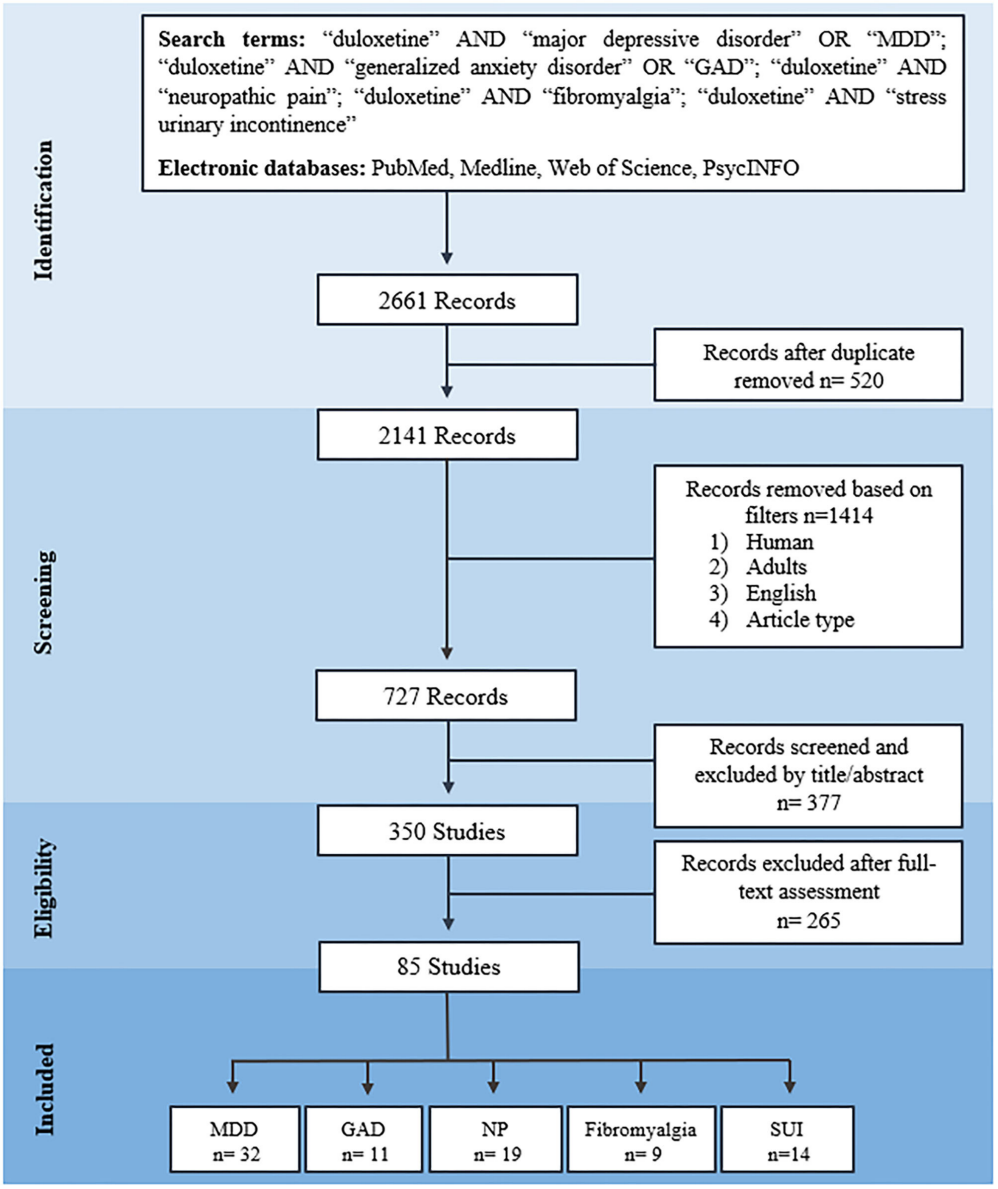
PRISMA 2009 flow diagram of search process. MDD, major depressive disorder; GAD, generalized anxiety disorder; NP, neuropathic pain; SUI, stress urinary incontinence.

The first stage of the searching process comprised a search in the electronic databases using specific search terms, where 2,661 reports were identified. In the second stage, inclusion criteria were applied, duplicate reports were removed, and 727 records by title and abstract were studied. Three hundred and forty-two studies were analyzed in the third stage, and their full-text versions were carefully examined. In this stage, they were 85 eligible studies that met the inclusion criteria and the JBI recommendations (Supplementary Tables 1–4). Finally, in the fourth stage, studies on the different clinical conditions for which duloxetine treatment is approved −32 studies on major depressive disorder (MDD), 11 studies on GAD, 19 studies on NP, 9 studies on fibromyalgia, and 14 studies on SUI were selected (Figure 1).

### Study Characteristics

**Figure 2:**
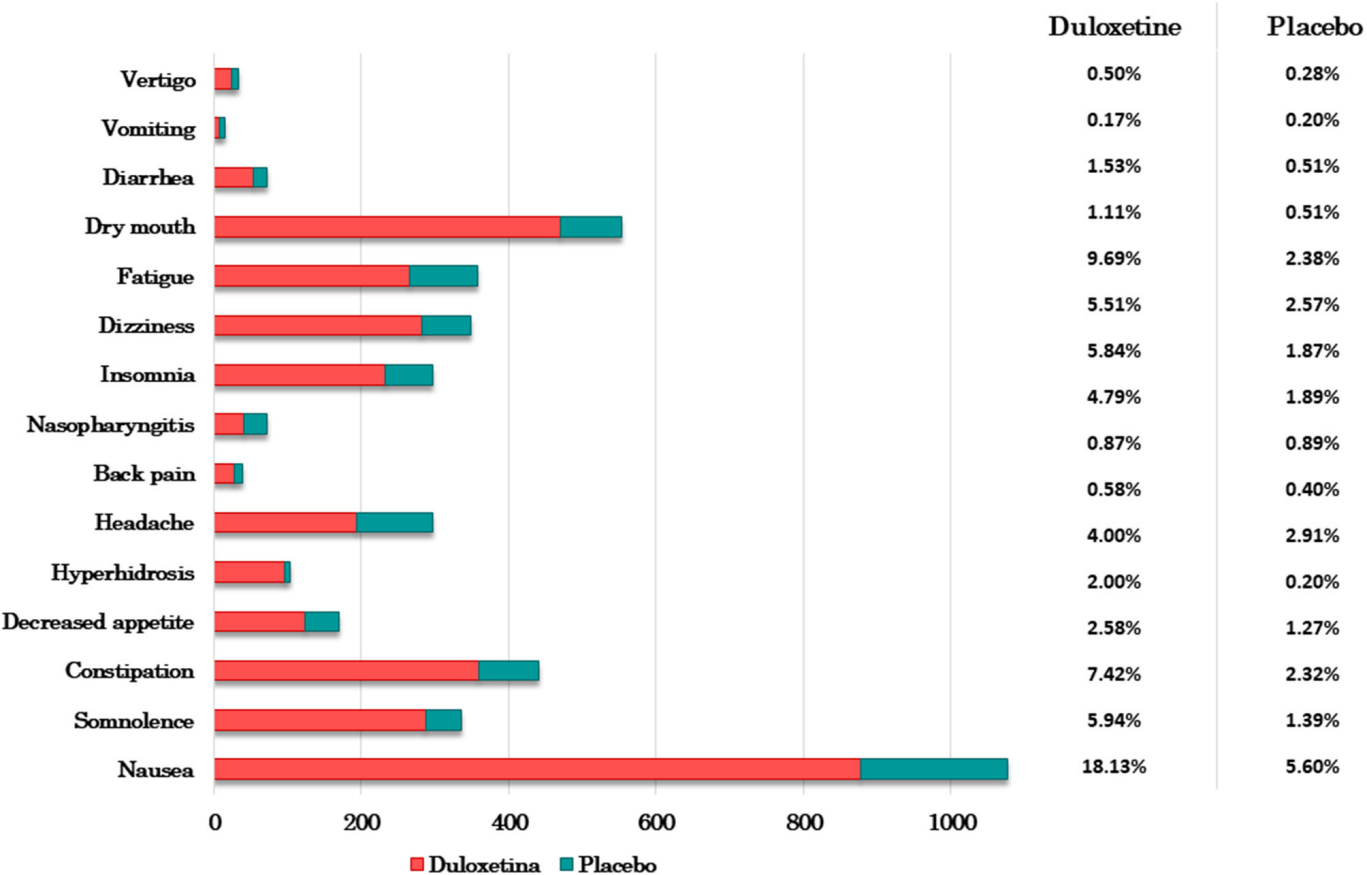
Adverse events of duloxetine-treated patients and placebo patients. Bar graph of the most common adverse effects vs. the number (N) of participants who developed them (N_total_ duloxetine-treated patients = 4,848 and N_total_ placebo patients = 3,536). The table shows the corresponding percentage.

The eligible studies were examined and categorized by clinical condition. Five clinical conditions were considered for the treatment with duloxetine: MDD, GAD, NP, FMS, and SUI. Thus, eighty-five studies were scrutinized and a total of 34,808 participants were enrolled (25,448 female, 9,108 male; and 581 participants that in their study were not differentiated by gender), with an age range of 18–97 years. The studies included 21,406 patients that were treated with duloxetine with a dose ranged from 20 to 120 mg and a treatment duration of 12 ± 14.39 weeks. The main reasons of dropout were adverse events (59.5%), lack of effectiveness (20.3%), patient's decision (9.5%), loss of follow-up (5.4%), non-adherence to treatment (2.7%), hospitalizations (1.3%), and others (1.3%). Within adverse events, the most common were nausea (18.13%), dry mouth (9.69%), constipation (7.42%), and somnolence (5.94%) (Figure 2). Cardiovascular disease was an exclusion criterion of 7% of the studies, and cardiovascular adverse events (hypertension, tachycardia, myocardial ischemia, increased blood pressure, and arrythmia) were evaluated in 68.2% of the studies, where 49.4% reported statistical insignificance for these TEAEs (*P* <0.05), and 11.8% showed a correlation between elevated heart rate and duloxetine treatment. Regarding the type of studies, 58.7% of the studies are RCTs, 25.9% are cohort studies, 11.8% are quasi-experimental studies (non-randomized) and 3.5% are case-control studies.

#### Major Depressive Disorder

MDD studies comprised 1,836 patients that were treated with 20–120 mg of duloxetine during 8 ± 17.05 weeks. This condition was diagnosed based on the Diagnosis and Statistical Manual of Mental Disorders (DSM) from their third to fifth edition. Efficacy of duloxetine was measured in 78.1% of the studies using the Hamilton Depression Rating Scale (HAMD), the Geriatric Depression Scale (GDS), the Montgomery and Asberg Depression Rating Scale (MADRS) or the Brief Pain Inventory (BPI) when pain, and MDD were concomitant. Safety of treatment with duloxetine was assessed in 25% of the studies based on TEAEs, and tolerability was evaluated in 31.3% of studies (35–66). Twenty-six studies were able to demonstrate the superiority of duloxetine over placebo or other antidepressants such as sertraline, fluvoxamine, venlafaxine, paroxetine, escitalopram, fluoxetine, and bupropion. Five studies did not find statistical significance (*P* ≥ 0.05) regarding the correlation between duloxetine and the outcomes and one study did not obtain significant results when comparing duloxetine with sertraline. Safety and tolerability were evaluated by TEAEs and the most common adverse events (AEs) were nausea, somnolence, dry mouth, hyperhidrosis, constipation, and sedation; patient's dropout rate was ~10% (Figure 2). This result was not significant in most of studies, concluding that duloxetine was safe and well-tolerated (see Table 1).

**Table 1:** Characteristics of the selected studies and included in the systematic review.

**References**	**N^**°**^ participants**	**Gender**	**Years (mean + SD)**	**Dose duloxetine**	**Treatment duration**	**Diagnosis scales, measures**	***P*-value**	**G Hedges**	**Type of study**	**Outcome**
**Major depressive disorder**										
De Donatis et al. (66)	n° duloxetine = 66 Total n°= 66	40 F/26 M	56.42 ± 14.55	60 mg	12 weeks	DSM-IV, HAMD21, serum concentration	*P* < 0.001	1.907	Cohort study	Treatment response MDD Serum concentration duloxetine
Mowla et al. (65)	n° duloxetine = 26 n° sertraline = 28 Total n°= 54	32 F/22 M	42.3	40–60 mg	6 weeks	DSM-V, SCID-I, HAMD21, CGI-2	*P* = 0.463	0.391	RCT double-blind	Compare the effects of sertraline with duloxetine in MDD HAMD21
Buoli et al. (64)	n° escitalopram = 10 n° citalopram = 19 n° paroxetine = 23 n° mirtazapine = 8 n° fluoxetine = 13 n° clomipramine = 8 n° sertraline = 14 n° trazodone = 6 n° duloxetine = 10 n° venlafaxine = 12 n° fluvoxamine = 12 n° amitriptyline = 10 n° bupropion = 5 Total n°= 150	115 F/35 M	51.03 ± 13.83	65.50 ± 15.89 (mg)	96 weeks	DSM-V, SCID-I	*P* < 0.01	2.984 (fluvoxamine) 3.623 (bupropion)	Clinical trial	Efficacy at long-term treatment of MDD Breslow's test
Robinson et al. (63)	n° duloxetine = 204 n° placebo = 95 Total n°= 299	191 F/108 M	73.01 ± 6.26	60–120 mg	24 weeks	DSM-IV, HAMD17, GDS, CGI-S, PGI-I, BPI, NRS, TEAEs	*P* = 0.004	4.545	RCT double-blind	Efficacy in elderly patients with MDD GDS
Martinez et al. (60)	n° duloxetine = 372 n° SSRIs = 378 Total n°= 750	536 F/214 M	44.3 ± 13.0	30–60 mg	12 weeks	DSM-IV, QIDS-SR, HAMD17, BPI, SDS	*P* < 0.01	4.250	RCT non-blinded	Efficacy in moderate-to-severe depressive episode HAMD17 total
Oakes et al. (61)	n° duloxetine = 261 n° placebo = 131 Total n°= 392	256 F/136 M	44.7 ± 12.2	60 mg	8 weeks	DSM-IV, HAMD17, SDS, SASS, CGI-S	*P* < 0.001	6.577	RCT double-blind phase IV	Efficacy HAMD17
Rosso et al. (62)	n° duloxetine = 25 n° bupropion = 21 Total n°= 46	31 F/15 M	47.6 ± 12.6	120 mg	6 weeks	DSM-IV, HAMD17, CGI-I, GAF	*P* = 0.793	0.076	RCT double-blind	Efficacy HAMD17
Brecht et al. (48)	n° duloxetine 60 = 167 n° duloxetine 120 = 171 Total n°= 338	251 F/87 M	44.8 ± 13.3	60–120 mg	8 weeks	DSM-IV, MADRS, HDRS-6, CGI-S, TEAEs	*P* = 0.88 TEAEs >10%	0.019	RCT double-blind	Efficacy and safety MADRS
Gaynor et al. (59)	n° duloxetine = 262 n° placebo = 266 Total n°= 528	302 F/226 M	46.2 ± 13	60 mg	8 weeks	DSM-IV, MINI, MADRS, BPI, SDS, CGI-S, PGI, TEAEs	*P* < 0.001 TEAEs = 5%	6.167	RCT double-blind	Efficacy and tolerability MADRS
Sagman et al. (58)	n° duloxetine responders = 115 n° duloxetine non-responders = 91 Total n°= 242	182 F/60 M	44.9 ± 12.5	60–120 mg	8 weeks	DSM-IV, BPI-SF, HAMD17	*P* = 0.042	-	Clinical trial open-label	Switching to duloxetine treatment BPI-SF
Herrera-Guzmán et al. (56)	n° duloxetine = 37 n° escitalopram =36 n° control = 37 Total n°= 110	78 F/32 M	33.21 ± 8.61	60 mg	24 weeks	DSM-IV, MINI, HAMD17, WAIS III, SWM, RVIP, MTS, Stroop test, ID/ED, SOC	*P* < 0.001	4.864	Case-control study	Efficacy in improving attention and executive functions HAMD17
Volonteri et al. (57)	n° duloxetine = 45 Total n°= 45	29 F/16 M	59.6 ± 12.79	30–120 mg	12 weeks	DSM-IV, HRSD, CGI-S, BDI, VAS, AEs	*P* < 0.001 AEs = 9%	9.402	Naturalistic open-label study	Clinical response and tolerability HRSD
Perahia et al. (44)	n° duloxetine = 146 n° placebo = 142 Total n°= 288	206 F/82 M	47.1 ± 12.8	60–120 mg	52 weeks (maintenance phase)	DSM-IV, MINI, HAMD17, CGI-S, PGI-I, SDS, VAS, SF-36, SQ-SS, TEAEs	*P* < 0.001 *P*_TEAEs_ > 0.05	5.380	RCT double-blind	Recurrence of MDD Safety and tolerability HAMD17
Karp et al. (53)	n° duloxetine = 40 Total n°= 40	26 F/14 M	74.4 ± 7.0	120 mg	16 weeks	DSM-IV, SCID, MMSE, HAMD17, UKU, AEs	*P* < 0.01 AEs = 12.5%	0.029	Open label Cohort study	Tolerability UKU
Kornstein et al. (54)	n° duloxetine non-remitters 60 = 130 n° duloxetine non remitters 120 = 118 n° duloxetine remitters 60 = 193 Total n°= 441	275 F/166 M	44.7 ± 12.77	30–120 mg	16 weeks	DSM-IV, HAMD17, IDS-C-30, QIDS-C-16, BPI-SF, VAS, CGI-S, PGI, TEAEs	*P* ≤ 0.05	8.8858.491	RCT double-blind	Efficacy HAMD17
Perahia et al. (43)	n° duloxetine direct switch = 183 n° duloxetine start-taper switch = 185 Total n°= 368	283 F/85 M	49.05 ± 12.8	60–120 mg	8 weeks	DSM-IV, HAMD17, CGI-S, EQ-5D, VAS, SQ-SS, SF-36, TEAEs	*P* ≤ 0.001 *P* ≤ 0.001	–	RCT open-label non-inferiority study	Efficacy and tolerability HAMD17
Perahia et al. (42)	n° duloxetine = 330 n° venlafaxine = 337 Total n°= 667	450 F/217 M	44.3 ± 12.8	60–120 mg	12 weeks	DSM-IV, MINI, HAMD17, HAMA, CGI-S, PGI-I, TEAEs	*P* = 0.440	1.084	RCT double-blind	Global benefit–risk HAMD17
Raskin et al. (51)	n° duloxetine = 207 n° placebo = 104 Total n°= 311	185 F/146 M	72.6 ± 5.7	60 mg	8 weeks	DSM-IV, HAMD17	*P* < 0.001 *P* = 0.014 *P* = 0.042	2.355 (dry mouth) 2.091 (Nausea) 2.219 (Diarrhea)	RCT double-blind	Safety and tolerability Adverse effects
Volpe (55)	n° duloxetine = 30 Total n°= 30	28 F/2 M	41 ± 8	60 mg	8 weeks	DSM-IV, MADRS, VAS, WHOQoL-BREF, AEs	*P* < 0.001 AEs = 6.7%	2.874	Open label Cohort study	Efficacy and tolerability MADRS
Brecht et al. (46)	n° duloxetine = 162 n° placebo = 165 Total n°= 327	241 F/86 M	48.1	60 mg	8 weeks	DSM-IV, MINI, MADRS, BPI-SF, CGI-S, TEAEs	*P* = 0.0008 TEAEs>10%	–	RCT double-blind	Efficacy and safety BPI-SF
Lee et al. (47)	n° duloxetine = 238 n° paroxetine = 240 Total n°= 478	333 F/145 M	39.0 ± 13.95	60 mg	8 weeks	DSM-IV, HAMD17, VAS, CGI-S, PGI-I, TEAEs	*P* = 0.218 *P*_TEAEs_ > 0.05	-	RCT double-blind	Efficacy and safety HAMD17
Pigott et al. (49)	n° duloxetine = 273 n° escitalopram = 274 n° placebo = 137 Total n°= 684	446 F/238 M	41.1 ± 11.6	60–120 mg	8 months	DSM-IV, MINI, MADRS, CGI-S, PGI-I, HAMA, CSFQ, AEs	*P* = 0.44 AEs = 12.8%	0.774 3.272	RCT double-blind	Efficacy, safety, and tolerability HAMD17
Raskin et al. (50)	n° duloxetine = 207 n° placebo = 104 Total n°= 311	185 F/126 M	72.6 ± 5.7	60 mg	8 weeks	DSM-IV, HAMD17, MMSE, CGI-S, WAIS-III, VAS, CCS, GDS	*P* < 0.02	–	RCT double-blind	Efficacy on cognition, depression, and pain CCS
Wise et al. (52)	n° duloxetine = 828 n° placebo = 416 Total n°= 1,244	740 F/504 M	72.8 ± 5.6	60 mg	8 weeks	DSM-IV, MMSE, CCS, GDS, HAMD17, CGI-S, VAS, SF-36, TEAEs	*P* = 0.013 TEAEs = 9.7%	–	RCT double-blind	Safety and tolerability with comorbidities CCS
Fava et al. (45)	n° duloxetine 60 QD = 58 n° duloxetine 60 BID = 29 Total n°= 87	69 F/18 M	43.8 ± 11.17	60–120 mg	12 weeks	DSM-IV, HAMD17, CGI-S, VAS	*P* < 0.001	0.465	RCT double-blind	Depression relapses HAMD17
Perahia et al. (41)	n° duloxetine = 136 n° placebo = 142 Total n°= 278	202 F/76 M	45.7 ± 12.69	60 mg	26 weeks	DSM-IV, MINI, HRSD17, CGI-S	*P* ≤ 0.05	0.675	RCT double-blind	Relapse prevention Relapses
Perahia et al. (40)	n° duloxetine 40 BID = 93 n° duloxetine 60 BID = 103 n° placebo = 99 n° paroxetine = 97 Total n°= 392	273 F/119 M	45.43 ± 11.37	80–120 mg	8 weeks	DSM-IV, MINI, HAMD17, CGI-S, MADRS, HAMA, VAS	*P* ≤ 0.05	2.600 3.200 2.200	RCT double-blind	Efficacy HAMD17
Burt et al. (39)	n° duloxetine = 55 n° placebo = 59 Total n°= 114	114 F	47.7	60 mg	9 weeks	DSM-IV, HAMD17, CGI-S, PGI-I, VAS, SSI, QLDS	*P* < 0.001	0.686	RCT double-blind	Efficacy in women HAMD17
Goldstein et al. (36)	n° duloxetine 20 BID = 86 n° duloxetine 40 BID = 91 n° placebo = 89 n° paroxetine = 87 Total n°= 353	217 F/136 M	41 ± 11	40–80 mg	8 weeks	DSM-IV, HAMD17, VAS, CGI-I, PGI-I, QLDS	*P* = 0.002 *P* = 0.034 *P* = 0.285	–	RCT double-blind	Improvement of emotional and painful physical symptoms HAMD17
Detke et al. (37)	n° duloxetine = 123 n° placebo = 122 Total n°= 245	163 F/82 M	42.44 ± 13.74	60 mg	9 weeks	DSM-IV, MINI, HAMD17, CGI-S, PGI-I, QLDS, AEs	*P* < 0.001 AEs = 13.8%	–	RCT double-blind	Efficacy and tolerability HAMD17
Goldstein et al. (38)	n° duloxetine = 70 n° fluoxetine = 33 n° placebo = 70 Total n°= 173	111 F/6 3M	42.3 ± 10.8	40–120 mg	8 weeks	DSM-IV, MINI, HAMD17, CGI-S, MADRS, PGI, HAMA, AEs	*P* = 0.009 AEs = 4.3%	–	RCT double-blind	Efficacy, safety, and tolerability HAMD17
Berk et al. (35)	n° duloxetine = 93 Total n°= 93	62 F/31 M	38	20 mg	6 weeks	DSM-III, HAMD17, CGI-I, PGI,	−16.4 ± 6.7 (change)	2.565	Open label uncontrolled trial	Efficacy HAMD17
**Generalized depressive disorder**										
Alaka et al. (67)	n° duloxetine = 151 n° placebo= 140 Total n°= 291	226 F/65 M	71.4 ± 5.4	30–120 mg	10 weeks	DSM-IV, HAMA, SDS, HADS, CGI-I, TEAEs	*P < * 0.001 TEAEs = 9%	6.461	RCT double-blind	Efficacy and safety HAMA
Bodkin et al. (68)	n° duloxetine = 216 n° placebo = 213 Total n°= 429	257 F/172 M	45.0 ± 13.2	60–120 mg	26 weeks	DSM -IV, HAMA, CGI-I, MINI, HADS, SDS, SQ-SS, VAS	*P =* 0.028 *P < * 0.001	1.097 1.650	RCT double-blind	Relapses HAMA-1, VAS
Pierò et al. (69)	n° duloxetine = 23 n° escitalopram = 20 Total n°= 43	31 F/12 M	35.3 ± 17.4	60 mg	26 weeks	DSM-IV, HAMA, HDRS, CGI, GAF	*P < * 0.001	0.374	Clinical trial non-randomized	Effectiveness of 6-months treatment with escitalopram and duloxetine HAMA
Wu et al. (70)	n° duloxetine = 108 n° placebo = 102 Total n°= 210	106 F/104 M	37.3 ± 11.9	60–120 mg	15 weeks	DSM-IV, CAS, RDS, CGI-S, SDS, HADS-A, HAMA, TEAEs	*P =* 0.006 *P*_TEAEs_ < 0.05	0.237	RCT double-blind phase III	Efficacy, tolerability, and safety HADS-A
Nicolini et al. (71)	n° duloxetine 20 = 158 n° duloxetine 60-120 = 158 n° venlafaxine = 169 n° placebo = 170 Total n°= 581	–	42.8	20–120 mg	10 weeks	DSM-IV, HAMA, HADS, CAS, CGI-I	*P ≤* 0.001	5.286	RCT double-blind	Symptoms improvement HAMA
Allgulander et al. (72)	n° duloxetine = 320 n° venlafaxine = 333 n° placebo = 331 Total n°= 984	596 F/388 M	41.6 ± 13.2	60–120 mg	10 weeks	DSM-IV, MINI, HADS, CAS, RDS, CGI-S	*P ≤* 0.001	–	RCT double-blind	Duloxetine vs. Venlafaxine efficacy HAMA
Davidson et la. (73)	n° duloxetine = 42 n° placebo = 28 Total n°= 70	38 F/42 M	70.1 ± 4.3	60–120 mg	9–10 weeks	DSM-IV, MINI, HAMA, HADS, CAS, RDS, CGI-S, TEAEs	*P =* 0.029 *P*_TEAEs_ <0.05	3.164	RCT double-blind	Efficacy and tolerability HAMA
Pollack et al. (74)	n° duloxetine = 668 n° placebo = 495 Total n°= 1,163	753 F/410 M	42.5 ± 13.3	60–120 mg	4 weeks	DSM-IV, HAMA, CGI-S, SDS	*P < * 0.001	–	RCT double-blind	Early improvement HAMA
Russell et al. (75)	n° duloxetine = 208 n° placebo = 146 Total n°= 354	247 F/107 M	42.1 ± 12.7	60–120 mg	12 weeks	DSM-IV, MINI, HAMA, VAS, HDAS, CAS, RDS, CGI-S	*P =* 0.017	–	RCT double-blind phase III	Efficacy HAMA
Rynn et al. (76)	n° duloxetine = 168 n° placebo = 159 Total n°= 327	202 F/125 M	42.2 ± 13.9	60–120 mg	10 weeks	DSM-IV, HAMA, CGI-S, HADS, CAS, RDS, TEAEs	*P =* 0.023 *P*_TEAEs_ <0.05	–	RCT double-blind	Efficacy and safety HAMA
Hartford et al. (77)	n° duloxetine = 162 n° venlafaxine = 164 n° placebo = 161 Total n°= 487	305 F/182 M	40.4 ± 13.6	60–120 mg	10 weeks	DSM-IV, SIGH-A, HADS, CAS, CGI-S, HAMA, TEAEs	*P ≤* 0.01 TEAEs = 5%	3.838 4.791	RCT double-blind phase II	Efficacy and tolerability HAMA
	Total n°= 487									
**Neuropathic pain**										
Salehifar et al. (78)	n° duloxetine = 42 n° pregabalin = 40 Total n°= 82	82 F	48.7 ± 9.63	30–60 mg	6 weeks	VAS, NCI-CTCAE v4.03, PNQ, AEs	*P < * 0.001	1.647 1.676 1.587	RCT double-blind phase II	Efficacy and safety of pregabalin and duloxetine in taxane-induced peripheral neuropathy VAS, NCI-CTCAE v4.03, PNQ
Jha et al. (79)	n° duloxetine = 9 n° pregabalin = 25 Total n°= 34	18 F/16 M	55.8 ± 8.59	20–30 mg	16 weeks	VAS, SF-MPQ, Mc-Gill, NRS, DN-4, AEs	*P < * 0.001	–	Cohort study	Efficacy, safety, and tolerability of pregabalin compared to duloxetine in DPNP Mc-Gill
Farshchian et al. (80)	n° duloxetine = 52 n° venlafaxine = 52 n° placebo = 52 Total n°= 156	124 F/32 M	57.4 ± 14.5	30 mg	4 weeks	RTOG criteria	*P < * 0.05	–	RCT double-blind	Effects of venlafaxine vs. duloxetine on chemotherapy-induced peripheral neuropathy
Schukro et al. (81)	n° duloxetine = 14 n° placebo = 11 Total n°= 25	21 F/20 M	57.9 ± 13.4	120 mg	4 weeks	VAS, pain DETECT questionnaire, BDI, SF-36	*P =* 0.001	0.675	RCT double-blind	Efficacy of duloxetine in low back pain with radicular pain VAS
Yasuda et al. (82)	n° duloxetine 40 = 129 n° duloxetine 60 = 129 Total n°= 258	62 F/196 M	60.1 ± 10.0	40–60 mg	52 weeks	BPI, PGI-I, AEs	*P < * 0.0001 AE ≤ 13.6%	3.273 3.473	RCT double-blind	Long-term efficacy and safety: duloxetine in diabetic neuropathic pain BPI
Gao et al. (83)	n° duloxetine = 203 n° placebo = 202 Total n°= 405	223 F/182 M	61.6 ± 9.7	60 mg	12 weeks	BPI-S, PGI-I, SDS, QIDS-SR, TEAEs	*P =* 0.030 TEAEs = 8.4%	3.071	RCT double-blind phase III	Efficacy and safety: duloxetine in diabetic neuropathic pain BPI-S
Happich et al. (84)	n° duloxetine = 931 n° pregabalin = 248 n° gabapentin = 351 Total n°= 1,530	794 F/736 M	64.0 ± 11.66	60 mg	36 weeks	BPI, CGI, PGI, HADS, SF-12, SDS	*P =* 0.029 *P < * 0.001	0.175 0.531	Cohort study	The effectiveness of duloxetine in DPNP BPI
Irving et al. (85)	n° duloxetine = 138 n° pregabalin = 134 n° duloxetine + gabapentin = 135 Total n°= 407	165 F/242 M	60.9 ± 10.2	60 mg	12 weeks	TEAEs, LSEQ, CSFQ, TEAEs	*P >* 0.05 P_TEAEs_= 0.04	0.172 0.187	RCT open-label non-inferiority study	Safety and tolerability in DPNP TEAEs
Vollmer et al. (86)	n° duloxetine = 118 n° placebo = 121 Total n°= 239	179 F/60 M	50.8 ± 9.7	30–60 mg	6 weeks	DSM-IV, MINI, C-SSRS, BDI-II, CGI-S, BPI, MS-QoL-54, PGI-I, MFIS, TEAEs	*P =* 0.001 TEAEs = 13.6%	4.606	RCT double-blind	Efficacy and tolerability neuropathic pain in multiple sclerosis API
Smith et al. (87)	n° duloxetine = 109 n° placebo = 111 Total n°= 220	138 F/82 M	60 ± 10.4	60 mg	12 weeks	BPI-SF	*P =* 0.003	0.513	RCT double-blind phase III	Effects of duloxetine on chemotherapy-induced peripheral neuropathy BPI-SF
Tesfaye et al. (88)	n° duloxetine = 401 n° pregabalin = 403 n° duloxetine + pregabalin = 339 Total n°= 1,143	514 F/629 M	61.5 ± 10.62	60 mg	20 weeks	BPI-MSF, BDI-II	*P =* 0.370	0.539	RCT double-blind	Efficacy DPNP BPI-MSF
Boyle et al. (89)	n° duloxetine = 28 n° pregabalin = 27 n° amitriptyline = 28 Total n°= 83	26 F/57 M	65.1 ± 8.9	60-120 mg	4 weeks	BPI-S, SF-36, PSG	*P < * 0.05	0.500 1.000	RCT double-blind	Impact on pain, polysomnographic sleep, daytime functioning, and quality of life in DPNP BPI-S
Tanenberg et al. (90)	n° duloxetine = 138 n° pregabalin = 134 n° duloxetine + gabapentin = 135 Total n°= 407	165 F/242 M	60.9 ± 10.2	60 mg	12 weeks	BPI, BDI-II, PGI-I, SDS, TEAEs, Pain rating	*P =* 0.08	1.000	RCT open-label non-inferiority study	Duloxetine is non-inferior to (as good as) pregabalin in DPNP Pain rating
Skljarevski et al. (91)	n° duloxetine QD = 115 n° duloxetine = 216 Total n°= 331	134 F/197 M	62.6 ± 9.4	60–120 mg	26 weeks	Pain rating, BPI	*P =* 0.017	2.562	RCT open-label	Effect of duloxetine 60 mg QD in patients with DPNP Pain ratings
Armstrong et al. (92)	n° duloxetine QD = 344 n° duloxetine BID = 341 n° placebo = 339 Total n°= 1,024	572 F/452 M	59.7 ± 10.7	60 mg QD 60 mg BID	12 weeks	DSM-IV, MINI, SF-36, BPI, EQ-5D	*P =* 0.004 *P < * 0.001	10.00 10.00	RCT double-blind	Efficacy in DPNP EQ-5D index
Wernicke et al. (93)	n° duloxetine = 197 n° routine care = 96 Total n°= 293	158 F/135 M	58.1 ± 10.5	120 mg	52 weeks	DSM-IV, MNSI, TEAEs, SF-36, EQ-5D, TEAES	*P < * 0.01 TEAEs = 5.6%	4.020	RCT open-label	Safety at a fixed-dose of 60 mg BID in DPNP SF-36
Raskin et al. (94)	n° duloxetine BID = 334 n° placebo QD = 115 Total n°= 449	215 F/234 M	59.8 ± 10.6	120 mg QD 60 mg BID	28 weeks	BPI, CGI-S, MNSI, TEAEs	*P =* 0.020 TEAEs <5%	0.229	RCT open-label	Safety and tolerability in diabetic neuropathy TEAEs
Goldstein et al. (95)	n° duloxetine 20 = 115 n° duloxetine 60 = 114 n° duloxetine 120 = 113 n° placebo = 115 Total n°= 457	176 F/281 M	60.1 ± 10.9	20–120 mg	12 weeks	DSM-IV, MINI, MNSI, 24-h Average Pain Score, BPI-S, AEs	*P >* 0.05 *P ≤* 0.01 *P ≤* 0.001 AEs <20%	1.000 3.667 4.791	RCT double-blind	Efficacy and safety in diabetic neuropathy 24-h Average Pain Score BPI-S
Raskin et al. (96)	n° duloxetine QD = 116 n° duloxetine BID = 116 n° placebo = 116 Total n°= 348	186 F/162 M	58.8 ± 10.1	60–120 mg	12 weeks	DSM-IV, MINI, MNSI, 24-h Average Pain Score, TEAEs	*P < * 0.001 TEAEs = 12.1%	5.000 4.833	RCT double-blind	Efficacy and safety in DPNP 24-h average pain score
**Fibromyalgia**										
Murakami et al. (97)	n° duloxetine = 50 n° placebo = 71 Total n°= 121	99 F/22 M	47.3 ± 11.9	20–60 mg	48 weeks	BPI, PGI-I, CGI-I, FIQ, BDI-II, SF-36, AEs	*P < * 0.05 AEs 10.1% (moderate AE)	0.159	RCT open-label, phase III	Efficacy and safety BPI
Murakami et al. (98)	n° duloxetine = 191 n° placebo = 195 Total n°= 386	321 F/65 M	48.7 ± 11.9	60 mg	14 weeks	BPI, FIQ, SF-36, BDI-II	*P =* 0.5456	0.061	RCT double-blind phase III	BPI change average
Mohs et al. (99)	n° duloxetine = 80 n° placebo = 76 Total n°= 156	144 F/12 M	21–88	60–120 mg	24 weeks	BPI, DSM-IV, VLRT, SDST, TMT	*P >* 0.05	0.065	RCT double-blind	Cognition effectiveness BPI
Mease et al. (33)	*Study 1: total = 278* n° duloxetine 120 = 79 n° duloxetine 60/120 = 127 n° placebo/Dlx = 72 *Study 2: total: 204* n° duloxetine 60 = 17 n° duloxetine 120=82 n° duloxetine 60/120 = 2 n° placebo/duloxetine 60 = 103	Study 1: 267 F/11 M Study 2: 194 F/10 M	52.0 ± 9.6	60–120 mg	28 weeks	BPI, PGI-I, BDI-II, HDRS, SF-36	*Study 1:* *P < * 0.001 *Study 2:* *P =* 0.580	*Study 1:* 0.297 0.406 *Study 2:* 0.247 0.055 0.000	2 RCT double-blind phase II	Risk/benefit profile for duloxetine BPI
Arnold et al. (100)	n° duloxetine = 263 n° placebo = 267 Total n°= 530	244 F/19 M	50.7 ± 11.3	60–120 mg	24 weeks	BPI, CGI-S, BDI, SF-36, DSM-IV, BAI, CPFQ, MFI	*P < * 0.001	4.000	RCT double-blind	Symptoms improvement BPI
Chappell et al. (101)	n° duloxetine 60 = 104 n° duloxetine 120 = 203 Total n°= 307	293 F/14 M	49.0 ± 11.07	60–120 mg	52 weeks	BPI, FIQ, PGI-I, CGI-S, SDS, AEs	*P ≤* 0.05 AEs = 21.1%	1.843	RCT double-blind phase III	Efficacy and safety FIQ
Russell et al. (102)	n° duloxetine = 376 n° placebo = 144 Total n°= 520	356 F/20 M	51.3 ± 10.9	20–120 mg	24 weeks	BPI, PGI-I, AEs	*P ≤* 0.05 AEs = 18.0%	3.953 2.619	RCT double-blind	Efficacy and safety BPI
Arnold et al. (103)	n° duloxetine = 240 n° placebo = 118 Total n°= 358	240 F	49.6 ± 10.9	60–120 mg	12 weeks	BPI, FIQ, CGI-S, PGI-I, HDRS, QLDS, SF-36, SDS, TEAEs	*P < * 0.01 TEAEs = 18.7%	5.721 5.768	RCT double-blind	Efficacy and safety BPI
Arnold et al. (104)	n° duloxetine = 104 n° placebo = 103 Total n°= 207	92 F/12 M	49.9 ± 12.3	120 mg	12 weeks	FIQ, CGI-S, PGI-I, DSM-IV, BDI-II, BAI, SF-36, QLDS, SDS, TEAEs	*P =* 0.027 P_TEAEs_= 0.229	3.115	RCT double-blind	Efficacy and safety FIQ
**Stress urinary incontinence**										
Cornu et al. (105)	n° duloxetine = 16 n° placebo = 15 Total n°= 31	31 M	68.3 ± 6.9	80 mg	12 weeks	I-QoL, IIQ-SF, UDI-SF, USPQ, ICIQ-SF, BDI-II, IEF, TEAEs	*P < * 0.0001 P_TEAEs_= 0.27	1.735	RCT double-blind	Efficacy and safety IEF
Cardozo et al. (106)	n° duloxetine = 1,378 n° placebo = 1,380 Total n°= 2,758	2,758 F	55.51 ± 11.77	80 mg	6 weeks	IEF, PGI-I, KHQ, TEAEs	*P < * 0.001 TEAEs = 21%	0.235	RCT double-blind	Efficacy and safety IEF
Bent et al. (107)	n° duloxetine = 300 n° placebo = 288 Total n°= 588	588 F	53.2 ± 12.5	80 mg	8 weeks	IEF, ICIQ-SF, I-QOL, PGI-I, TEAEs	*P < * 0.001 TEAEs = 21.3%	-	RCT double-blind	Efficacy and safety IEF
Lin et al. (108)	n° duloxetine = 61 n° placebo = 60 Total n°= 121	121 F	56.31 ± 11.0	80 mg	8 weeks	IEF, I-QOL, PGI-I, TEAEs	*P < * 0.001 TEAEs = 26.7%	-	RCT double-blind	Efficacy and safety IEF
Schagen et al. (109)	n° duloxetine = 131 n° placebo = 134 Total n°= 165	165 F	70.63 ± 5.08	40–80 mg	12 weeks	IEF, PGI-I, I-QOL, BDI-II, AEs	*P < * 0.001 P_TEAEs_= 0.210	-	RCT double-blind phase IV	Efficacy and safety in community-dwelling women ≥65 years IEF
Castro-Diaz et al. (110)	n° duloxetine 20 BID = 133 n° duloxetine 40 QD = 127 n° duloxetine 40 BID = 136 n° placebo = 120 Total n°= 516	516 F	53.0 ± 10.6	20-80 mg	8 weeks	IEF, ICIQ-SF, I-QOL, PGI-I, TEAEs	*P =* 0.008 TEAEs = 11.8%	0.331 0.436 0.611	RCT double-blind	Effect of dose escalation on the tolerability and efficacy TEAEs
Schlenker et al. (111)	n° duloxetine BID = 20 Total n°= 20	20 M	65.6	80 mg	1–35 weeks	SUIQ, AEs	*P < * 0.001 TEAEs = 30%	0.655	Cohort study	Efficacy and safety for men with stress incontinence (use off-label) Average daily use of incontinence pads
Weinstein et al. (112)	n° duloxetine BID = 2,960 Total n°= 2,960	2,960 F	49.6	80 mg	10 weeks	SUIQ, I-QOL, PGI-S, BDI-II, IEF, AEs	*P < * 0.05 AEs = 24.3%	0.186 0.113 Comparation with Caucasian subgroup	RCT open-label phase III	Efficacy and safety in racial and ethnic subgroups I-QOL
Ghoniem et al. (113)	n° duloxetine = 52 n° PFMT = 50 n° no PFMT = 47 n° combined = 52 Total n°= 201	201 F	53	80 mg	12 weeks	SUI, IEF, I-QOL, PGI-I	*P < * 0.001	0.043	RCT double-blind	Efficacy of duloxetine alone or combined with PFMT IEF
Kinchen et al. (114)	n° duloxetine BID = 224 n° placebo = 227 Total n°= 451	451 F	52.7 ± 13.0	80 mg	12 weeks	I-QOL, PGI-I	*P =* 0.07	-	RCT double-blind	Effectiveness in improving quality of life I-QOL
Cardozo et al. (115)	n° duloxetine = 55 n° placebo = 54 Total n°= 109	109 F	54.5 ± 9.7	80–120mg	8 weeks	I-QOL, IEF	*P =* 0.003	0.545	RCT double-blind	Efficacy I-QOL
Millard et al. (116)	n° duloxetine BID = 227 n° placebo = 231 Total n°= 458	458 F	53.7	80 mg	12 weeks	SUI, IEF, I-QOL, PGI-I, PGI-S, AEs	*P =* 0.007 AEs = 17.2%	0.236	RCT double-blind	Efficacy and safety I-QOL
Van Kerrebroeck et al. (117)	n° duloxetine BID = 247 n° placebo = 247 Total n°= 494	494 F	52.0 ± 11	80 mg	12 weeks	IEF, I-QOL, PGI-I, PGI-S, TEAEs	*P =* 0.008 TEAEs = 22%	-	RCT double-blind	Efficacy and safety I-QOL
Dmochowski et al. (118)	n° duloxetine BID = 344 n° placebo = 339 Total n°= 683	683 F	52.3 ± 10.4	80 mg	12 weeks	SUI, IEF, I-QOL, PGI-I, BDI-II, TEAEs	*P < * 0.001 TEAEs = 24%	0.332	RCT double-blind	Efficacy and safety IEF, I-QOL

*MDD, Major Depressive Disorder; DSM-IV, Diagnostic and Statistical Manual of Mental Disorders; HAMD, Hamilton Rating Scale for Depression; SCID, Structured Clinical Interview for DSM; CGI-I, Clinical Impressions of Improvement; RCT, Randomized Controlled Trial; GDS, Geriatric Depression Scale; CGI-S, Clinical Global Impression of Severity; PGI-I, Patient's Global Impressions of Improvement; BPI, Brief Pain Inventory; NRS, Numeric Rating Scale; TEAEs, Treatment-emergent adverse events; QD, Once Daily; BID, Twice Daily; QIDS-SR, Quick Inventory of Depressive Symptomatology-Self-Rated; SDS, Sheehan Disability Scale; SSRIs, Selective Serotonin Reuptake Inhibitors; SASS, Social Adaptation Self-evaluation Scale; GAF, Global Assessment of Functioning Scale; MADRS, Montgomery-Asberg Depression Rating Scale; HDRS, Hamilton Depression Rating Scale; MINI, Mini International Neuropsychiatric Interview; BPI-SF, Brief Pain Inventory—Short Form; WAIS, Wechsler Adult Intelligence Scale; SWM, Spatial Working Memory; RVIP, Rapid Visual Information Processing; MTS, Match to Sample Visual Search; ID/ED, Intra–Extra-Dimensional Set Shift; SOC, Stockings of Cambridge; BDI, Beck Depression Inventory; VAS, Visual Analog Scale; AEs, Adverse Events; SF-36, Medical Outcomes Study 36-item short-form health survey; SQ-SS, Symptom Questionnaire, Somatic Subscale; MMSE, Mini-Mental State Examination; UKU, Udvalg for Kliniske Undersogelser-Committee of Clinical Investigations Side Effect Rating Scale; IDS-C-30, 30- item Inventory of Depressive Symptomatology-Clinician Rated; EQ-5D, European QOL Questionnaire-5 Dimension; HAMA, Hamilton Anxiety Rating Scale; WHOQoL-BREF, World Health Organization Quality of Life Scale; CSFQ, Changes in Sexual Functioning Questionnaire; CCS, Composite Cognitive Score; QLDS, Quality of Life in Depression Scale; HADS, Hospital Anxiety and Depression Scale; CAS, Covi Anxiety Scale; RDS, Raskin Depression Scale; SIGH-A, Structured Clinical Interview Guide for Hamilton Anxiety Rating Scale; NCI-CTCAE v4.03, National Cancer Institute Common Terminology Criteria for Adverse Events version 4.03; PNQ, Patient Neurotoxicity Questionnaire; SF-MPQ, Short form of the McGill Pain Questionnaire; McGill, Pain Questionnaire; DN-4, Neuropathic Pain Diagnostic Questionnaire; RTOG, Radiation Therapy Oncology Group; C-SSRS, Columbia Suicide Severity Rating Scale; QOL, Quality of Life; LSEQ, Leeds Sleep Evaluation Questionnaire; DPNP, Diabetic Peripheral Neuropathic Pain; API, Average Pain Intensity; MS-QoL-54, Multiple Sclerosis Quality of Life-54 Instrument; MFIS, Modified Fatigue Impact Scale; BPI-MSF, Brief Pain Inventory Modified Short Form; PSG, Polysomnographic; MNSI, Michigan Neuropathy Screening Instrument; FIQ, Fibromyalgia Impact Questionnaire; VLRT, Verbal Learning and Recall Test; SDST, Symbol Digit Substitution Test; TMT, Trail-Making Test; BAI, Beck Anxiety Inventory; CPFQ, Cognitive and Physical Functioning Questionnaire; MFI, Multidimensional Fatigue Inventory; I-QoL, Incontinence Quality of Life; PFMT, Pelvic Floor Muscle Training; IIQ-SF, Incontinence Impact Questionnaire Short Form; UDI-SF, Urogenital Distress Inventory Short Form; USPQ, Urinary Symptom Profile Questionnaire; ICIQ-UI-SF, Incontinence Questionnaire-Urinary Incontinence Short Form; IEF, Incontinence Episode Frequency; KHQ, King's Health Questionnaire; SUI, Stress Urinary Incontinence; SUIQ, Stress/Urge Incontinence Questionnaire*.

#### Generalized Anxiety Disorder

Eleven studies focused on GAD were included in this systematic review, which involved 2,608 patients treated with 20–120 mg of duloxetine with an average duration of treatment of 10 ± 6.59 weeks. All studies used the DSM to accomplish the diagnosis. Clinical evidence was based on the correlation between the Hamilton Anxiety Rating Scale (HAMA) and the outcomes. Therefore, all studies found statistical significance (*P* ≤ 0.05) when measured the efficacy (90.9% of the studies), safety and tolerability (both 27.3% of the studies) (67–77). TEAEs were measured and nausea, dry mouth, dizziness, and somnolence were reported as the most frequent AEs (Figure 2). One study reported suicidal ideation, although no statistical significance was found between duloxetine and placebo groups (69). Duloxetine was more effective, safe and tolerated than placebo or other antidepressants (escitalopram and venlafaxine) (Table 1).

#### Neuropathic Pain

The selected NP studies reported the use of duloxetine in the treatment of peripheral neuropathy induced by chemotherapy, diabetic peripheral neuropathic pain (DPNP), radiculopathy and neuropathic pain associated with multiple sclerosis (NP-MS) (Table 1). NP condition was diagnosed using specific criteria of pain detection, being the BPI the most commonly applied instrument. TEAEs were the measure for safety and tolerability. The dose of duloxetine applied was ranged from 20 to 120 mg during 12 ± 14.53 weeks. The core of the studies focused on 4,627 patients with NP, where the efficacy, safety, and tolerability of duloxetine were compared with placebo, anticonvulsant treatments (pregabalin and gabapentin), other antidepressants (venlafaxine and amitriptyline), or even with different daily doses of duloxetine. Thus, 78.9% of the studies reported efficacy outcomes, 47.4 and 21.1% of the studies described safety and tolerability of duloxetine, respectively (78–96). Three studies did not show statistical significance regarding efficacy, safety, and tolerability of duloxetine against anticonvulsants (*P* ≥ 0.05). Nausea, somnolence, insomnia, constipation, and decreased appetite were the most prevalent TEAEs (Figure 2). A minority of patients discontinued the treatment with duloxetine due to AEs (12.2%). Nevertheless, 84.2% of the studies supported the evidence of duloxetine as first-line treatment of NP conditions.

#### Fibromyalgia

Nine studies focusing on FMS were assessed and eligible. They involved a total of 1,918 patients. The average duration of treatment was 24 ± 13.78 weeks and the dose of duloxetine oscillated from 20 to 120 mg per day (Table 1). The BPI scale and Fibromyalgia Impact Questionnaire (FIQ) were the instruments employed to analyse the outcomes. Most studies (77.8%) evaluated the efficacy of duloxetine, and 55.6% provided data of the treatment safety (97–104, 119). Statistically significant results were obtained in seven studies, where duloxetine improved symptomatology, reducing the pain impact registered by BPI. In contrast, two studies reported no statistical differences relative to BPI change average and cognitive improvement in fibromyalgia patients. The duloxetine treatment was related to ~17% of adverse effects. Taking all these studies into account, duloxetine showed to be safe and effective in FMS treatment.

#### Stress Urinary Incontinence

Fourteen studies that involved 6,395 patients (99.5% female and 0.5% male) were assessed. Duloxetine doses were between 20 and 120 mg per day. Treatment duration was 12 ± 6.72 weeks and the Incontinence Episode Frequency (IEF) and Incontinence Quality of Life (I-QoL) were the instruments to measure the outcomes. All studies evaluated efficacy of duloxetine; 71.4 and 7.1% of the studies measured safety and tolerability, respectively (105–118). Treatment was discontinued in 22.1% of patients regarding TEAEs, being the most common fatigue, nausea, constipation, and dry mouth (Figure 2). These AEs tend to improve and disappear with continuing duloxetine therapy. In conclusion, significant results were found in all studies, proving the efficacy, safety, and tolerability of duloxetine in the treatment of SUI.

### Quality Assessment

A systematic review summarizes the evidence of the relevant literature, however, an unbiased search of studies without an explicit assess strategy could lead to a poor scientific report. The relevance and quality of the studies selected and included in this systematic review fulfilled the PRISMA recommendations (120), and JBI critical appraisal guidelines. The JBI is an evidence-based organization that develops strategies to conduct and perform a high quality systematic reviews (121). Thus, the quality determination of the studies included indicates that our research minimizes the risk of selection bias. Furthermore, a good systematic review relies on the studies it contains. Therefore, the inclusion of RCTs and clinical trials reduce the probability of bias due to their strict methodology (122).

## Discussion

In the last years, mutual pathophysiological mechanisms have been identified in depression, pain, and anxiety (11). Neuropathic pain, specifically DPNP, coexists with mental disorders, predominantly with depression and anxiety (123). Highlighting the functional impairment as a result of unremitting pain symptoms, neuropathy has been correlated with an increased risk of depression (124). On the other hand, the widespread spontaneous pain is the most debilitating symptom of FMS, that might be a link to depression and anxiety disorders as comorbid conditions (125). Due to the similar pathophysiologic mechanisms and high occurrence of FMS and depression, these clinical conditions were considered under a common approach to affective disorders, GAD and PTSD (19). Urinary incontinence is also a severe problem that affects 15–30% of adults over 60 years, and several studies have reported a link between urinary incontinence, anxiety and depression in women (22, 126).

The core of the pathophysiology of these clinical conditions is mostly due to the disruption of 5-HT and norepinephrine (NE) pathways (19, 22, 127). The monoaminergic hypothesis is based on a partial or total deficit of 5-HT or NE in the central nervous system (CNS) (128, 129). Somatic symptoms such as muscle tension, neuropathic and musculoskeletal pain, fatigue, or dizziness are common in MDD and GAD among other psychiatric disorders as result of aberrant 5-HT and NE neurotransmission (130). Regarding pain, antinociception and pronociceptive modulation occurs through 5-HT receptors, in both the central and peripheral nervous systems (131). As in pain, SUI involves the action of monoaminergic system. Evidence demonstrates that endogenous regulation of serotoninergic and noradrenergic mechanism in the spinal cord works simultaneously to maintain the reflex of urinary continence (132).

Therefore, the pharmacological treatment of clinical conditions with similar pathophysiology involves a global perception of coexisting disorders. In this sense, antidepressants such as duloxetine have been considered effective in the treatment of MDD, GAD, NP, FMS, and SUI (133). Duloxetine is a serotonin-norepinephrine reuptake inhibitor, that is, a potent inhibitor of 5-HT transporter (SERT) and norepinephrine transporter (NET) (134). Due to this dual mechanism, its profile seems to have a different response compared to selective 5-HT reuptake inhibitors (SSRIs) (135). *In vivo* studies, duloxetine presented preferential inhibition of 5-HT reuptake and low affinity for hitamine-H1, alpha-1-norepinephrine, 5-HT_(1A, 1B, 1D)_, muscarinic acetylcholine, and opioid receptors (136). Clinically, duloxetine has been approved for diverse clinical conditions, acquiring new evidence over the years, also being prescribed to treat other neuropathic pain conditions and chronic musculoskeletal pain (80, 137).

In this systematic review, we considered the efficacy, safety, and tolerability of duloxetine in the treatment of current approved clinical conditions. Firstly, an individual search by clinical condition was achieved based on specific inclusion criteria. This strategy allowed us to find consistent results and objectively evaluate the outcomes. Concerning efficacy, duloxetine demonstrated effectiveness in over 80% of cases. However, some TEAEs are frequent, such as dry mouth, somnolence, nausea, constipation or hyperhidrosis, tending to decrease in time and disappear with continuing duloxetine therapy. Cardiovascular adverse events, such as hypertension, increased heart rate, myocardial ischemia, are also associated with duloxetine administration (29). Within these, only the increase in heart rate was statistically significant, although not being clinically relevant. In sum, duloxetine was considered in all assessed reports as a safe and well-tolerated treatment even in cardiovascular disease, as well as in elderly patients (29, 51, 82, 93). In this sense, our results prove that duloxetine is an option with a valid and consolidated therapeutic value. Secondly, we focused on the clinical conditions' comorbidity. The coexistence of depression, anxiety, and pain is a frequent state, as well as, depression and SUI, and FMS and depression. Therefore, the treatment with duloxetine is largely used due to its dual mechanism that ameliorate the symptoms associated with the concomitant clinical conditions (e.g., MDD and NP). Moreover, we observed a strong link between MDD and pain. This correlation suggests a bidirectional pattern: MDD could be a predictor of chronic pain which in turn might predict the persistence of MDD (138). Thirdly, although the dropout rate with duloxetine treatment reaches around 20% in certain cases, similar rates were found in placebo and other antidepressants or anticonvulsants treatments. Finally, some considerations should be taken into account regarding to duloxetine prescription and titration. Alcohol, tobacco and coffee (caffeine) are the most widely consumed psychoactive substances worldwide (139, 140). Evidence suggests that plasmatic serum levels of duloxetine were decreased (about 15%) in smoking patients due to the induction of CYP1A2 (141). Hepatotoxicity was also observed in patients whose alcohol consumption was significant (142). Lastly, caffeine is also metabolized by CYP1A2, like duloxetine, and this may increase duloxetine serum levels. However, this interference needs more supporting evidence.

We performed this systematic review in order to include as much evidence as possible. In this process, we analyzed a large number of studies to support our conclusions. Nevertheless, we found some limitations. The inclusion criteria exclude reports in a language other than English. Thus, significant studies may have been missed with this strategy. Our research protocol dismisses qualitative and pooled integrative data analysis of RCTs to avoid repeated analysis of RCTs data and qualitative findings duplication. Regarding the effect size of studies, some of them did not report sufficient statistical data to compute Hedges's g (e.g., standard deviation). However, we decided to include these 19 studies due to their relevance respect to the evaluated outcomes.

In conclusion, there is a substantial amount of evidence in support of efficacy, safety, and tolerability of duloxetine in the treatment of MDD, GAD, NP, FMS, and SUI. The dose range of 60–120 mg daily demonstrated efficacy in most of the studies assessed. TEAEs were mild to moderate, and AEs decreased or remitted with continuing duloxetine treatment. Treatment discontinuation due to both AEs and ineffectiveness of duloxetine yielded enough acceptable results to conclude that duloxetine is safe and well-tolerated. In addition, duloxetine is a monotherapy approach that might be useful to treat concomitant disorders with parallel pathophysiological pathways such as MDD and NP, which is an advantage for patients (avoiding polytherapy issues) and a successful cost-effective alternative.

## Data Availability Statement

All datasets generated for this study are included in the article/Supplementary Material.

## Author Contributions

Data analysis and the first draft of the manuscript was written by DR-A and TR-B. All authors contributed to the study design, acquired data to analysis, and read and approved the final manuscript.

## Conflict of Interest

JO declares paid positions, honoraria or being part of advisory boards by Angelini, AstraZeneca, Bristol-Myers, Casen Ricordati, Esteve, GSK, Janssen, Lilly, Lundbeck, Novartis, Otsuka, Pfizer and Sanofi, and grants from Ministry of Health Spanish National Institute of Heath Carlos III, National Substance Abuse Plan, and Galician Innovation Programs (GAIN) in the last 10 years. The remaining authors declare that the research was conducted in the absence of any commercial or financial relationships that could be construed as a potential conflict of interest.
