# Supplementary material for: A Systematic Review of Efficacy, Safety, and Tolerability of Duloxetine
Source: Front Psychiatry. 2020 Oct 23;11:554899. doi: 10.3389/fpsyt.2020.554899 (PMC7644852; doi:10.3389/fpsyt.2020.554899)
Supplement: Supplementary file 1 [file Data_Sheet_1.pdf]

Supplementary table 1: Quality assessment by *JBI Critical Appraisal Checklist* for Randomized Controlled Trial (RCT) included in the systematic review.

	JBI Checklist for RCT												
	**Q1**	**Q2**	**Q3**	**Q4**	**Q5**	**Q6**	**Q7**	**Q8**	**Q9**	**Q10**	**Q11**	**Q12**	**Q13**
Fibromyalgia													
**Murakami et al. 2017**	Y	Y	Y	N	N	N	Y	Y	N	Y	Y	Y	Y
**Murakami et al. 2015**	Y	Y	Y	Y	Y	N	Y	Y	N	Y	Y	Y	Y
**Mohs et al., 2012**	Y	Y	Y	Y	Y	N	Y	Y	N	Y	Y	Y	Y
**Mease et al., 2010**	Y	Y	Y	Y	Y	N	Y	Y	N	Y	Y	Y	Y
**Arnold et al., 2010**	Y	Y	Y	Y	Y	N	Y	Y	Y	Y	Y	Y	Y
**Chappel et al., 2009**	Y	Y	Y	Y	Y	N	Y	Y	Y	Y	Y	Y	Y
**Russell et al., 2007**	Y	Y	Y	Y	Y	N	Y	Y	Y	Y	Y	Y	Y
**Arnold et al., 2005**	Y	Y	Y	Y	Y	N	Y	Y	Y	Y	Y	Y	Y
**Arnold et al., 2004**	Y	Y	Y	Y	Y	N	Y	Y	Y	Y	Y	Y	Y
Generalized Anxiety Disorder													
**Alaka et al., 2014**	Y	Y	Y	Y	Y	Y	Y	Y	Y	Y	Y	Y	Y
**Bodkin et al., 2011**	Y	Y	Y	Y	Y	N	Y	Y	N	Y	Y	Y	Y
**Wu et al., 2011**	Y	Y	Y	Y	Y	N	Y	Y	Y	Y	Y	Y	Y
**Nicoli et al., 2009**	Y	Y	Y	Y	Y	N	Y	Y	Y	Y	Y	Y	N
**Allgulander et al., 2008**	Y	Y	Y	Y	Y	N	Y	Y	Y	Y	Y	N	N
**Davidson et la., 2008**	Y	Y	Y	Y	Y	N	Y	Y	N	Y	Y	Y	Y
**Pollack et al., 2008**	Y	Y	Y	Y	Y	N	Y	Y	N	Y	Y	N	N
**Russell et al., 2008**	Y	Y	Y	Y	Y	N	Y	Y	Y	Y	Y	N	N
**Rynn et al., 2008**	Y	Y	Y	Y	Y	N	Y	Y	Y	Y	Y	N	N
**Harford et al., 2007**	Y	Y	Y	Y	Y	N	Y	Y	Y	Y	Y	Y	Y
Neuropathic Pain													
**Salehifar et al., 2020**	Y	Y	Y	Y	Y	N	Y	Y	Y	Y	Y	Y	Y
**Farshchian et al., 2018**	Y	Y	Y	Y	Y	N	Y	Y	N	N	N	N	N
**Schukro et al., 2016**	Y	Y	Y	Y	Y	N	Y	Y	Y	Y	Y	Y	Y
**Yasuda et al., 2016**	Y	Y	Y	Y	Y	N	Y	Y	N	Y	Y	Y	Y
**Gao et al., 2015**	Y	Y	Y	Y	Y	N	Y	Y	Y	Y	Y	Y	Y
**Irving et al., 2014**	Y	Y	Y	N	N	N	Y	Y	N	Y	Y	Y	Y
**Vollmer et al., 2014**	Y	Y	Y	Y	Y	N	Y	Y	Y	Y	Y	Y	Y
**Smith et al., 2013**	Y	Y	Y	Y	Y	N	Y	Y	Y	Y	Y	Y	Y
**Tesfaye et al., 2013**	Y	Y	Y	Y	Y	N	Y	Y	Y	Y	Y	Y	Y
**Boyle et al., 2012**	Y	Y	Y	Y	Y	N	Y	Y	N	Y	Y	Y	Y
**Tanenberg et al., 2011**	Y	Y	Y	N	N	N	Y	Y	Y	Y	Y	Y	Y
**Skljarevski et al., 2009**	Y	Y	Y	N	N	N	Y	Y	Y	Y	Y	Y	Y
**Armstrong et al., 2007**	Y	Y	Y	Y	Y	N	Y	Y	Y	Y	Y	Y	Y
**Wernicke et al., 2007**	Y	Y	Y	N	N	N	Y	Y	Y	Y	Y	Y	Y
**Raskin et al., 2006**	Y	Y	Y	N	N	N	Y	Y	Y	Y	Y	Y	Y
**Goldstein et al., 2005**	Y	Y	Y	Y	Y	N	Y	Y	Y	Y	Y	Y	Y
**Raskin et al., 2005**	Y	Y	Y	Y	Y	N	Y	Y	Y	Y	Y	Y	Y
Urinary Incontinence													
**Cornu et al., 2011**	Y	NA	Y	Y	Y	N	Y	Y	Y	Y	Y	Y	Y
**Cardozo et al., 2010**	Y	NA	Y	Y	Y	N	Y	Y	Y	Y	Y	Y	Y
**Bente t al., 2008**	Y	NA	Y	Y	Y	N	Y	Y	Y	Y	Y	N	N
**Lin et al., 2008**	Y	NA	Y	Y	Y	N	Y	Y	Y	Y	Y	N	N
**Schagen et al., 2008**	Y	NA	Y	Y	Y	N	Y	Y	Y	Y	Y	N	N
**Castro-Diaz et al., 2007**	Y	NA	Y	Y	Y	N	Y	Y	Y	Y	Y	Y	Y
**Weinstein et al., 2006**	Y	Y	Y	Y	Y	N	Y	Y	Y	Y	Y	Y	Y
**Ghoniem et al., 2005**	Y	Y	Y	Y	Y	N	Y	Y	Y	Y	Y	Y	Y
**Kinchen et al., 2005**	Y	NA	Y	Y	Y	N	Y	Y	Y	Y	Y	N	N
**Cardozo et al., 2004**	Y	NA	Y	Y	Y	N	Y	Y	Y	Y	Y	Y	Y
**Millard et al., 2004**	Y	NA	Y	Y	Y	N	Y	Y	Y	Y	Y	Y	Y
**Van Kerrebroeck et al., 2004**	Y	NA	Y	Y	Y	N	Y	Y	Y	Y	Y	N	N
**Dmochowski et al., 2003**	Y	NA	Y	Y	Y	N	N	Y	Y	Y	Y	Y	Y
Major Depressive Disorder													
**Mowla et al., 2016**	Y	Y	Y	Y	Y	N	Y	Y	N	Y	Y	Y	Y
**Robinson et al., 2014**	Y	Y	Y	Y	Y	N	Y	Y	Y	Y	Y	Y	Y
**Oakes et al., 2012**	Y	Y	Y	Y	Y	N	Y	Y	Y	Y	Y	Y	Y
**Rosso et al., 2012**	Y	Y	Y	Y	N	N	Y	Y	Y	Y	Y	Y	Y
**Brecht et al., 2011**	Y	Y	Y	Y	Y	N	Y	Y	N	Y	Y	Y	Y
**Gaynor et al., 2011**	Y	Y	Y	Y	Y	N	Y	Y	N	Y	Y	Y	Y

**Perahia et al., 2009**	Y	Y	Y	Y	Y	N	Y	Y	Y	Y	Y	Y	Y
**Kornstein et al., 2008**	Y	Y	Y	Y	Y	N	Y	Y	N	Y	Y	Y	Y
**Perahia et al., 2008b**	Y	Y	Y	Y	Y	N	Y	Y	Y	Y	Y	Y	Y
**Raskin et al., 2008**	Y	Y	Y	Y	Y	N	Y	Y	Y	Y	Y	Y	Y
**Brecht et al., 2007**	Y	Y	Y	Y	Y	N	Y	Y	N	Y	Y	Y	N
**Lee et al., 2007**	Y	Y	Y	Y	Y	N	Y	Y	Y	Y	Y	Y	N
**Pigott et al., 2007**	Y	Y	Y	Y	Y	N	Y	Y	Y	Y	Y	Y	Y
**Raskin et al., 2007**	Y	Y	Y	Y	Y	N	Y	Y	Y	Y	Y	Y	N
**Wise et al., 2007**	Y	Y	Y	Y	Y	N	Y	Y	Y	Y	Y	Y	N
**Fava et al., 2006**	Y	Y	Y	Y	Y	N	Y	N	Y	Y	Y	Y	Y
**Perahia et al., 2006a**	Y	Y	Y	Y	Y	N	Y	N	Y	Y	Y	Y	Y
**Perahia et al., 2006b**	Y	Y	Y	Y	Y	N	Y	Y	Y	Y	Y	Y	Y
**Burt et al., 2005**	Y	Y	Y	Y	Y	N	Y	N	Y	Y	Y	Y	Y
**Goldstein et al., 2004**	Y	Y	Y	Y	Y	N	Y	Y	Y	Y	Y	Y	N
**Detke et al., 2002**	Y	Y	Y	Y	Y	N	Y	N	Y	Y	Y	Y	N
**Goldstein et al., 2002**	Y	Y	Y	Y	Y	N	Y	N	Y	Y	Y	Y	N

Y- Yes; N- No; NA- Not applicable.

Supplementary table 2: Quality assessment by *JBI Critical Appraisal Checklist* for Quasi-Experimental Studies (QES) (non-randomized experimental studies) included in the systematic review.

	**JBI Checklist for QES**								
	**Q1**	**Q2**	**Q3**	**Q4**	**Q5**	**Q6**	**Q7**	**Q8**	**Q9**
Generalized Anxiety Disorder									
**Pierò et al., 2011**	Y	Y	Y	N	Y	Y	Y	Y	Y
Major Depressive Disorder									
**Buoli et al., 2015**	Y	Y	Y	N	Y	Y	Y	Y	Y
**Sagman et al., 2011**	Y	Y	Y	N	Y	Y	Y	Y	Y
**Perahia et al., 2008a**	Y	Y	Y	N	Y	Y	Y	Y	Y

Y- Yes; N- No.

Supplementary table 3: Quality assessment by *JBI Critical Appraisal Checklist* for Cohort Studies included in the systematic review.

	**JBI Checklist for Cohort**										
	**Q1**	**Q2**	**Q3**	**Q4**	**Q5**	**Q6**	**Q7**	**Q8**	**Q9**	**Q10**	**Q11**
Neuropathic Pain											
**Jha et al., 2019**	Y	Y	Y	N	N	Y	Y	N	Y	NA	N
**Happich et al., 2014**	Y	Y	Y	Y	Y	N	Y	Y	Y	Y	Y
Urinary Incontinence											
**Schlenker et al., 2006**	NA	NA	Y	Y	Y	Y	Y	Y	Y	Y	Y
Major Depressive Disorder											
**De Donatis et al., 2019**	NA	NA	Y	Y	Y	N	Y	Y	Y	NA	Y
**Volonteri et al., 2010**	NA	NA	Y	N	N	U	Y	Y	Y	NA	Y
**Perahia et al., 2009**	NA	NA	Y	Y	Y	Y	Y	Y	Y	NA	Y
**Karp et al., 2008**	NA	NA	Y	Y	Y	Y	N	Y	Y	NA	Y
**Volpe, 2008**	NA	NA	Y	Y	Y	Y	N	Y	Y	NA	Y
**Berk et al., 1997**	NA	NA	Y	Y	Y	Y	Y	Y	Y	NA	Y

Y- Yes; N- No; U- Unclear; NA- Not applicable.

Supplementary table 4: Quality assessment by *JBI Critical Appraisal Checklist* for Case Control Studies included in the systematic review.

	JBI Checklist for Case-Control									
	**Q1**	**Q2**	**Q3**	**Q4**	**Q5**	**Q6**	**Q7**	**Q8**	**Q9**	**Q10**
Major Depressive Disorder										
**Herrera-Guzmán et al., 2010**	Y	Y	Y	Y	Y	Y	N	Y	Y	Y

Y- Yes; N- No.
